# Donor human milk: the influence of processing technologies on its nutritional and microbial composition

**DOI:** 10.3389/fnut.2024.1468886

**Published:** 2024-11-01

**Authors:** Ruth Conboy-Stephenson, R. Paul Ross, Alan L. Kelly, Catherine Stanton

**Affiliations:** ^1^Teagasc Food Research Centre, Moorepark, Fermoy, Co. Cork, Ireland; ^2^School of Microbiology, University College Cork, Cork, Ireland; ^3^APC Microbiome Institute, University College Cork, Cork, Ireland; ^4^School of Food and Nutritional Sciences, University College Cork, Cork, Ireland

**Keywords:** donor human milk, Holder pasteurization, non-thermal, bioactive compounds, high-pressure processing, ultraviolet-C irradiation, microwave heating, high-temperature short-time

## Abstract

Human milk is regarded as the gold standard nutrition for newborn infants, providing all nutrients required for adequate growth and development from birth to 6 months. In addition, human milk is host to an array of bioactive factors that confer immune protection to the newborn infant. For this reason, the supply of human milk is crucial for premature, seriously ill, or low birth weight infants (<1,500 g). When a mother’s own milk is unavailable, donor human milk is the recommended alternative by the World Health Organization. Prior to consumption, donor human milk undergoes pasteurization to ensure the eradication of bacterial agents and prevent the transfer of potentially pathogenic organisms. Currently, Holder Pasteurization, a heat-based treatment, is the widely adopted pasteurization technique used by milk banks. Holder pasteurization has demonstrated degradative effects on some of milk’s biologically active factors, thus depleting critical bioactive agents with known functional, protective, and beneficial properties, ultimately reducing the immunoprotective value of donor human milk. As a result, alternative strategies for the processing of donor human milk have garnered much interest. These include thermal and non-thermal techniques. In the current review, we describe the effects of Holder pasteurization and alternative milk processing technologies on the nutritional and bioactive properties of milk. In addition, the capacity of each technique to ensure microbial inactivation of milk is summarized. These include the most extensively studied, high-temperature short-time and high-pressure processing, the emerging yet promising techniques, microwave heating and UV-C irradiation, and the lesser studied technologies, thermoultrasonication, retort processing, pulsed electric field, and gamma irradiation. Herein, we collate the findings of studies, to date, to allow for greater insight into the existing gaps in scientific knowledge. It is apparent that the lack of a cohesive standardized approach to human milk processing has resulted in contrasting findings, preventing a direct comparative analysis of the research. We conclude that donor human milk is a unique and valuable resource to the health sector, and although substantial research has been completed, persistent data disparities must be overcome to ensure optimal nutrition for the vulnerable newborn preterm infant group, in particular.

## 1 Introduction

Milk is a unique species-specific fluid produced by mammals post-partum. Human milk is the established optimum nutrition for newborn infants, contributing to the newborn infant’s health and development with long-lasting effects across the lifespan. Human milk has been linked with reduced incidence of infections in infants, including necrotizing enterocolitis (NEC), diarrhea, and respiratory diseases, and is associated with reduced rates of infant morbidity and mortality (1–3). Human milk is a rich source of macro- and micronutrients, an array of bioactive components and microbes (4). The mammary gland confers the unique ability to adjust milk composition during lactation to the requirements of the growing infant. As a result, human milk composition varies based on a number of maternal and environmental factors, including gestational age, stage of lactation, stage of milk (fore vs. hind), age, diet, geographical location, and diurnal variations (5).

Although fresh mothers’ own milk is regarded as the optimal food for newborn infants (6), donor human milk (DHM) is recommended by the World Health Organization (WHO) when mothers’ own milk is not available (7, 8). DHM is particularly beneficial to infants born prematurely, with a very low birth weight (<1,500 g) or a serious illness (9, 10). Preterm and low birth weight infants are at increased risk of serious health complications, including NEC, due to an underdeveloped immune system (11, 12). For these infants, DHM contributes to the optimal recovery, growth, and health of the newborn and reduces the risk of negative medical outcomes (13–16). However, increasing evidence suggests that there is lower availability of favorable immunoprotective compounds in DHM than in fresh milk as a result of the collection, storage, and processing of DHM (17).

### 1.1 Milk banks—current handling practices and techniques

Human milk banks provide an essential service to infants who are unable to access their mothers’ own milk. Milk banks are responsible for the collection, processing, storage, and distribution of DHM. Currently, there are 282 human milk banks supplying DHM in Europe, with a further 18 planned (18). Similar to Europe, there has been an upsurge in the number of milk banks in the USA, with 11 banks in 2012 which increased to 32 as of 2023 (19). Furthermore, in the USA, a number of for-profit milk banks have also been established. However, for the purpose of this review, we will only focus on non-profit milk banks. As the number of milk banks increases, so too does the number of hospitals and patients using their services. An analysis of surveys conducted by the Centers for Disease Control and Prevention on Maternity Practices in Infant Nutrition between 2007 and 2011 demonstrated an increase in the use of DHM by intensive care units from 25.1% in 2007 to 45.2% in 2011 (20). Indeed, a 2015 survey notes a 74% increase in neonatal facilities using DHM compared with 2011 (9).

The women who become donors donate on a volunteer basis if they have excess milk supply. Donors undergo health evaluation including behavioral and serological screening to prevent the transfer of infectious diseases (21). Although no international legislation exists for donor screening and milk processing, a number of guidelines have been developed. The European Milk Banking Association has published recommendations on milk banking procedures; these recommendations align closely with those of the Human Milk Bank Association of North America, the National Institute for Health and Care Excellence (NICE), and The Perron Rotary Express Bank Australia (19, 22–25).

**Figure 1:**
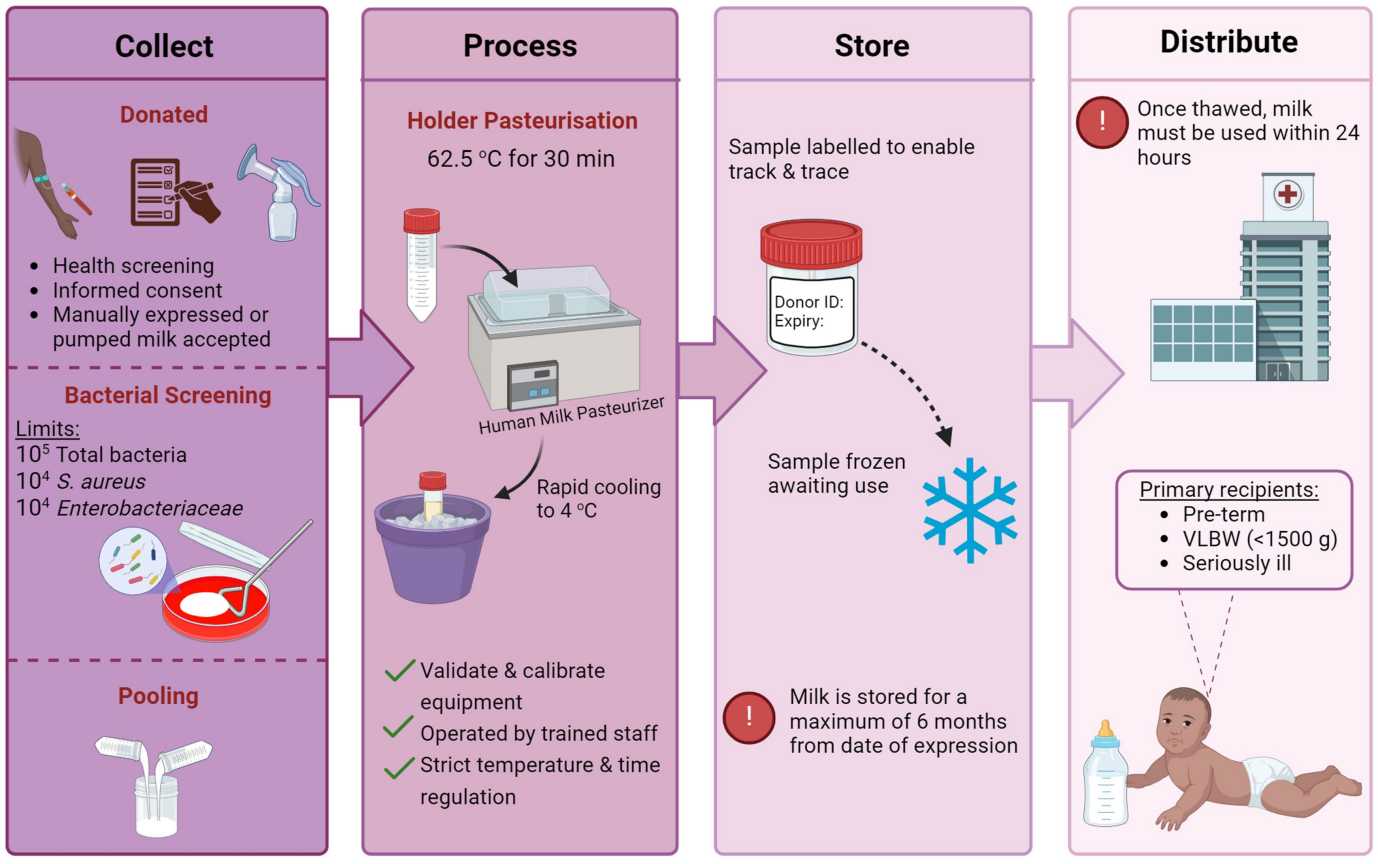
The handling and processing steps of human milk in a hospital milk bank setting [created with BioRender.com].

Following the donation, milk undergoes a series of processing steps including pooling, pasteurization, and bacteriological screening (see Figure 1) (26). For pooling, milk from multiple donors is thawed and pooled to create a uniform batch of donor milk with a relatively standard level of macronutrients. Although no official criteria exist regarding the number of donors per batch, Colaizy et al. (27) suggest that three to five donors per pool is sufficient to reduce variability and optimize macronutrient content. Once pooled, milk undergoes pasteurization and bacterial screening. The microbiological quality and safety of DHM are a primary concern for human milk banks. In addition to the naturally occurring microorganisms of breast milk, DHM is at risk of exogenous contamination during the handling and processing steps (28, 29).

Holder pasteurization (HoP), also known as low-temperature long-time pasteurization, is performed to ensure the inactivation of potentially harmful bacterial agents and viruses. HoP is the standard preservation technique adopted by milk banks worldwide (22), subjecting milk samples to a temperature of 62.5°C for 30 min. Following treatment, milk is rapidly cooled and frozen awaiting use. Current guidelines regarding bacterial screening by milk banks vary. NICE recommends that samples should be screened prior to pasteurization and discarded if total microbial counts exceed 10^5^ CFU mL^−1^ (24). Furthermore, counts of 10^4^ CFU/mL or higher of *Enterobacteriaceae* or *Staphylococcus aureus* are unacceptable. Post-pasteurization, no level of bacterial growth in milk is permissible.

Preterm infants are the primary recipients of DHM, highlighting the pivotal role of milk banks in healthcare settings (30). Current research endeavors to better understand the impact of handling, processing, and storage procedures on DHM composition and address any subsequent nutrient gap. In particular, optimizing the pasteurization method used to ensure minimal loss of bioactive factors of milk while preserving its microbial safety is desirable.

## 2 The effect of holder pasteurization on the composition of donor human milk

### 2.1 Macronutrient composition of human milk

Human milk is a tailor-made liquid food that provides complete nutrition to the developing infant (31). The nutritional constituents of milk originate from three key sources, the lactocytes, dietary origins, and maternal stores (32). The macronutrients in human milk constitute lipid, carbohydrate, and protein. Human milk composition consists of approximately 87% of water, 3.8% of fat, 7% of lactose, and 1% of protein (33, 34).

#### 2.1.1 Lipids

In milk, 98–99% of the lipid content is comprised of triglycerides in the form of saturated fatty acids (SFA), monounsaturated fatty acids (MUFA), and polyunsaturated fatty acids (PUFA) (16). Triglycerides are composed of a glycerol molecule with three fatty acids attached. These fatty acids can be combined in over 45 different positions and combinations (31). The remaining lipid portion of milk includes cholesterol, phospholipids, gangliosides, and sphingolipids, located within the milk fat globular membrane (16). The lipid content of human milk accounts for approximately 50% of milk’s energy supply and is linked with improved neurodevelopment, immune function, and metabolism in the infant (33, 35).

To date, several studies have investigated the effect of HoP on the lipid content of DHM. The majority of findings suggest the retention of DHM lipid content following HoP (36–42). Pitino et al. noted that the fatty acid profile of milk is preserved following HoP (43). Indeed, no modifications to the fatty acid composition of DHM were observed post-HoP (36, 38, 44–48). However, an increase in the proportion of medium-chain fatty acids alongside a reduction in oleic acid was reported (49). In contrast, some studies have reported reductions in the total lipid content of DHM of 3.5–8.9% (50–53) with a subsequent loss in total energy of 2.8–2.9% (50, 51). Similarly, an even greater decrease in fat and energy of 25 and 16%, respectively, was documented in DHM from a South Indian milk bank (54). In addition, Capriati et al. (55) noted a reduction in the triglyceride content of DHM of 21%. A reduction in the total lipid content of DHM is unexpected, and the reason for these reported reductions remains unclear.

#### 2.1.2 Carbohydrates

Human milk consists of many complex carbohydrates, of which the major and most studied are lactose and human milk oligosaccharides (HMOs). Lactose is a disaccharide sugar and is the main carbohydrate in milk (56), accounting for 40% of the energy supply (57) and 60–70% of the total osmolarity of human milk (16). In addition, lactose facilitates the absorption of minerals and is associated with the innate immune response (58, 59). Unlike other macronutrients, lactose levels remain relatively constant over 12 months of lactation, with a reported mean concentration of 61.4 g/L (60). HMOs are a group of structurally diverse unconjugated glycans unique to human milk (61). After lipids and lactose, they are the third most abundant solid component of breast milk, constituting 10–15 g/L of mature milk (62). Over 200 structures of HMOs have been identified in human milk to date (63). HMOs have an important role in the immune system due to their anti-adhesive properties against pathogenic microbes, their promotion of a *Bifidobacterium*-rich gut microbiome, and their modulation of intestinal cell responses (62, 64).

There is consensus regarding the effects of HoP on DHM carbohydrate levels. Adhisivam et al. observed no significant reduction in carbohydrate levels following HoP (54). Indeed, despite one report of a slight reduction (0.92%) in lactose levels (53), the stability of carbohydrate levels post-HoP has been further demonstrated elsewhere (37, 39–42, 50, 52, 65–67). Additionally, other milk carbohydrates were preserved following HoP, including myo-inositol (65, 66), glucose (51, 65, 66), glycosaminoglycans (68), and oligosaccharides (67, 69).

#### 2.1.3 Proteins

Accounting for just 1%, the protein content of human milk is low but has high bioavailability (70). Human milk proteins can be categorized into three main classes, whey protein, caseins, and mucin proteins (71). Whey protein is the predominant protein in milk, with a whey protein-to-casein ratio of 90:10 in colostrum and 60:40 in mature milk (72). Milk proteins contribute to a wide range of functions, including providing nitrogen and amino acids for tissue growth and maintenance, antimicrobial activity, immunomodulatory effects, and aiding digestion (34, 73). Overall, findings indicate that HoP does not reduce the total protein content of DHM. Indeed, a number of studies reported the retention of total protein levels post-HoP (37, 39–42, 50, 51, 74–76). However, similar to the lipid content, contrasting data on the effect of HoP on the total protein content of DHM have been reported. For instance, a reduction in protein levels following HoP of 12.5–13% was demonstrated (77, 54), with a less but still significant reduction of 2.5–3.9% also being reported (52, 53). Importantly, there have been reported changes to the protein profile of HoP-treated DHM in comparison with raw milk (76, 78). In particular, Silvestre et al. recorded a significant reduction in lysine levels of DHM following HoP. Lysine is an essential amino acid found in human milk and can be an indicator of the protein quality of milk. HoP caused a significant loss in the average available lysine of 29.3% when compared with untreated milk (75). These findings suggest that although HoP may preserve the total protein content, the protein quality of milk may be modified or even diminished. For instance, decreased lysine levels are often used as an indicator of early-stage Maillard reaction (79). Furthermore, a separate study noted increased production of three key Maillard reaction products, furosine, carboxymethyllysine, and N-epsilon-carboxyethyllysine, following HoP (80). Taken together, these findings suggest that HoP-treated DHM may have increased levels of Maillard reaction products, potentially affecting the nutritional value of donor milk. Future studies should investigate the effect of HoP on the protein quality, the potential formation of Maillard reaction products, and any subsequent health effects on the newborn infant.

Overall, despite the observed heterogeneity within the available data, studies to date, indicate the ability of HoP processing to preserve the total lipid, carbohydrate, and protein content of DHM. However, the potential modification of protein quality warrants further investigation. Future studies should endeavor to further elicit not only the effect on macronutrient concentration but also any subsequent consequences for the activity, quality, and bioavailability of nutritional constituents. The variability in findings may be due to the absence of a systematic approach to study design, sample characterization (sample volume, number of donors, and number of freeze–thaw cycles), and the analysis method. The sample storage history, mainly freezing, can result in alterations to certain fractions of milk, including the milk fat globule membrane, leaving components more vulnerable to thermal processing (27). Additionally, a high starting concentration of solid components may impact the efficacy of the analysis method. The use of older instruments or varying pasteurizer designs can increase the lag times for heating and cooling, altering the duration of thermal exposure for milk components. In particular, the choice of the analytical method appears to be a key influential factor, with studies choosing from a range of laboratory reference methods or commercially available infrared (IR) analyzers.

Milk banks primarily use IR analyzers to assess the nutritional composition of DHM. IR analyzers use near-, mid-, or Fourier transform IR spectroscopy. Recently, a mid-IR analyzer (Miris Human Milk Analyzer) was approved by the FDA and is available commercially (81). IR analyzers have the advantage of performing rapid analysis on low-volume milk samples. Perrin et al. confirmed that IR analyzers have a high degree of accuracy for crude protein and fat analysis (82). In contrast, carbohydrate determination was less precise, with Fourier- and mid-IR analysis reporting lower concentrations, while filtered- and near-IR led to increased levels (20–50%) when compared with reference values. These findings suggest that IR analysis is accurate at determining the protein and fat content but may not be the optimum choice for carbohydrate quantification. Furthermore, the correct calibration, validation, and operation of the instrument are critical to ensure consistent accuracy.

In addition to IR analyzers, laboratory-based analysis methods are still widely used and considered favorably. A review of laboratory techniques for macronutrient quantification has recently been performed (83), in addition to a review of methodologies for lactose analysis (57). These include the Kjeldahl or Dumas method for protein determination, a combination of solvent extraction and gravimetry for lipid analysis, and either high-performance lipid chromatography or high-performance anion exchange for carbohydrate quantification. Both study design and method of analysis have the potential to impact results, while a standardized choice of analytical method and predetermined selection criteria for the milk sample may reduce the inconsistencies noted in findings to date.

Overall, despite some contrasting findings, HoP appears to adequately preserve the macronutrient portion of DHM and provide high nutritive value to the infant.

### 2.2 Micronutrients

Micronutrients in milk refer to essential vitamins and minerals that aid human growth, development, and cell function (84). Vitamin and mineral intakes are linked with a variety of physiological functions, including ocular development, hormone synthesis, enzymatic and metabolic functioning, and neurodevelopment (16, 85).

Only a small number of studies have examined the effect of HoP on the mineral content of DHM. HoP did not cause a significant reduction in calcium (37, 55). Additionally, phosphorus, iron, copper, and zinc levels remained stable following HoP (37). However, a change in the distribution pattern of zinc following processing was observed, which could impact zinc bioavailability (37). Usually, zinc concentration is at its highest in the whey portion of milk with lower levels in the fat and casein fractions. Goes et al. demonstrated that although this was the case pre-processing, following pasteurization, the levels of zinc in the whey portion decreased and an increase occurred in the fat fraction of milk.

Findings indicate the potential stability of mineral concentrations post-processing. However, only a small number of minerals have been quantified. Future studies should expand to include a wider range of minerals, such as potassium, magnesium, and chloride. Furthermore, the consequences of any potential modifications to the distribution pattern of minerals and other nutrients as a result of pasteurization should be further examined.

Studies to date indicate a vulnerability of some water-soluble vitamins to thermal processing conditions of HoP. In particular, the degradation of vitamin C as a result of HoP leads to losses of up to 36% (44, 46, 86). Similarly, folacin and vitamin B6 underwent significant losses of 31 and 15%, respectively (86). In contrast, other studies reported the stability of both (74, 87). The variability in results is likely due to differing analytical techniques. The retention of riboflavin (B2), biotin (B7), niacin (B3), thiamine (B1), vitamin B2, vitamin B12, and pantothenic acid following HoP has been observed (74, 86, 87). It has been reported by some studies that fat-soluble vitamins, including vitamin A (37, 86), vitamin D (86), and vitamin E (44, 86) are robust to the processing conditions of HoP. However, a study by Ribeiro et al. noted a 34% reduction in vitamin A with high-performance liquid chromatography following pasteurization (88). A similar reduction of 32.5% in vitamin A was also reported (89). Vitamin E levels following HoP underwent a reduction of 17–25.5%, 13–47.3, and 33% in *α*-, *γ*- and *δ*-tocopherols, respectively (45, 46).

Potential changes to the vitamin concentration and bioavailability of DHM following processing are concerning as low birth weight infants, the primary recipients of donor milk, have increased vitamin requirements. Interestingly, vitamin A level analysis in both HoP-treated and untreated DHM suggests that neither supply adequate levels, providing only 43.6 and 66%, respectively, of the recommended nutrient requirement (88, 89). The clinical implications of reduced vitamin bioavailability in DHM require further consideration. Evidently, HoP has varying effects on the vitamin profile of DHM, which are dependent on the vitamins’ ability to withstand thermal exposure. Any future milk processing technique should endeavor to optimize micronutrient preservation.

### 2.3 Bioactive milk components

Human milk is a valuable source of bioactive compounds with a range of anti-inflammatory and antimicrobial properties that contribute to immune development and gut colonization. These include hormones, enzymes, cell signaling molecules, and bioactive agents such as immunoglobulins (Igs), lactoferrin, and lysozyme. A number of studies have indicated the negative influence of HoP on these bioactive compounds, mainly due to their proteinaceous structure.

#### 2.3.1 Immunoglobulins

There are five classes of Igs in human milk IgA, IgG, IgM, IgD, and IgE (90). The most abundant is IgA followed by IgG and IgM, with mean concentrations of 2.785, 0.059, and 0.036 g/L, respectively, across gestational age ranges (77). Indeed, secretory IgA (sIgA) accounts for 80–90% of the Ig profile of human milk (91). The Ig content of milk has a critical role in the protective capacity of human milk against infection (92).

The Ig profile of DHM has been widely studied, and findings suggest significant reductions in Ig levels as a result of HoP. Adhisivam and investigators reported a reduction in IgA of 30% (54). Similar reductions in IgA of >20% have been noted in a number of studies (77, 93–112) and even higher reductions of 50–60% have also been reported (65, 101, 102). Furthermore, numerous studies demonstrated a reduction in sIgA levels (74, 103–105). In addition, a decrease in IgG levels following HoP has been widely reported (96) although the extent of loss varied, from 23–34% (98, 106) to 60–79% (54, 77). All studies to date describe significant reductions in IgM concentration following HoP (65, 77, 96–98, 105). Overall, it is apparent that the processing conditions of HoP have severe degradative consequences on the Ig profile of DHM.

#### 2.3.2 Lactoferrin

Lactoferrin is a multifunctional glycoprotein of the transferrin family with enzymatic activity and antimicrobial, anti-inflammatory, and anti-infective properties (107, 108). Lactoferrin is best known for its role as an iron-binding protein. Indeed, lactoferrin has been shown to bind 20–45% of iron in human milk (108). Lactoferrin is the second most abundant protein in human milk (107). Concentrations of lactoferrin in milk vary according to the stage of lactation, with the highest levels in colostrum (7 g/L) and declining in mature milk (2–4 g/L) (109). The findings reported thus far indicate a degradative effect of HoP on the lactoferrin content of DHM. Mayayo et al. demonstrated that 80% of lactoferrin undergoes denaturation as a result of thermal exposure during HoP (110). Furthermore, the group speculate that lactoferrin degradation occurs as a result of protein aggregation caused by disulfide bonds. Separate investigations also noted a reduction in lactoferrin following HoP of 57–81% (93, 97, 101, 102, 105, 106). Goulding et al. (111) suggest that the heat stability of lactoferrin is influenced by environmental conditions including the degree of iron saturation, pH levels, and protein composition. Evidently, lactoferrin is poorly preserved during HoP, undergoing thermally induced degradation likely as a result of changes in the protein structure.

#### 2.3.3 Lysozyme

Lysozyme is a major enzyme in human milk with anti-infective properties (34), contributing to the bacteriostatic capacity of human milk. Findings to date demonstrate a reduction in the concentration of lysozyme following HoP (74, 101, 102). Koenig et al. noted reductions of 65–85% in colostrum (77). A similar decrease of 60.6% was reported in mature milk (93). Moreover, the effect on lysozyme activity was measured in a series of *Micrococcus lysodeikticus* turbidimetric and lyso-plate assays and determined reductions of 36–44% (98, 100, 112). Sousa et al. hypothesized that the loss of both lysozyme concentration and activity following HoP was due to the neutral pH environment of milk (7–7.4) (98). Indeed, lysozyme is heat stable at acidic pH (3–4) but heat labile at pH >7 (113). It is apparent that the thermal processing conditions of HoP, likely in combination with the pH environment of milk, result in lysozyme inactivation.

#### 2.3.4 Human milk enzymes

Human milk lipase activity consists of two enzymes, bile salt-stimulated lipase (BSSL) and lipoprotein lipase (LPL). BSSL facilitates the digestion and absorption of fat (114, 115). LPL participates in the production of milk lipids in the mammary gland (116), and although it has no known function in milk, it is thought to have a protective role during cold storage (117).

The evidence thus far indicates the near complete destruction of BSSL following HoP (47, 118, 119). In a study by Hamprecht et al. (74) <1% of lipase activity was retained. Similarly, Henderson et al. (38) noted a 100% reduction in LPL and BSSL activity. Comparable findings were reported by Kontopodi et al. in two separate studies (101, 102); the group noted low retention of BSSL levels (2–3%) and activity (4–7%) following HoP. The inactivation of BSSL as a result of thermal processing is not surprising. For instance, Pan et al. indicated that BSSL remained stable when exposed to 45°C for 30 min (120). However, following an increase in temperature, the stability of BSSL was significantly reduced at 50°C and completely destroyed at 60°C (120). It can be concluded that HoP treatment results in the denaturation of BSSL. BSSL is particularly important for newborn infants, due to their limited secretion of pancreatic lipase and bile salts. Indeed, pasteurization of DHM results in a reduction of lipid absorption of 30% in preterm infants (113). Discernibly, a loss of BSSL interferes with the lipid metabolism of the neonate and is a serious limitation of HoP.

Fewer studies have assessed the impact of HoP on other enzymes, including amylase and alkaline phosphatase (ALP); however, reports indicate their reduction. Amylase contributes to the digestion of starch; its presence in human milk negates the limited salivary and pancreatic amylase activity in the first few months of life (121). Similar to milk lipases, amylase is relatively heat-labile (113). However, amylase appears more resistant to HoP processing with a reduction of 6–15% (38, 122). ALP, due to its thermal resistance, is often used as a marker of adequate pasteurization (123, 124). Indeed, two studies noted the complete inactivation of ALP post-HoP (74, 102). Evidently, thermal exposure during pasteurization induces structural modifications, which can result in a loss of enzyme activity, functionality, and concentration. The degree of denaturation is dependent on the optimum conditions of the enzyme.

#### 2.3.5 Cell signaling molecules

Cell signaling molecules in DHM are important regulators of the inflammatory and immune response. Cell signaling molecules include cytokines, chemokines, and growth factors (GF). The influence of HoP on a variety of cytokines has been assessed with inconsistent results, see Table 1. The majority of findings to date suggest interleukin (IL)-2, IL-4, IL-5, IL-12, and IL-13 are not influenced by HoP (45, 49, 65); however, contrasting results have been noted (99, 125). It appears that HoP has no effect on IL-17 (45, 65). Both macrophage inflammatory protein (MIP)-1β and erythropoietin (EPO) were reduced by HoP (65, 126). Similarly, a reduction in IL-1β IL-6, IL-10, and tumor necrosis factor (TNF)-*α* was observed (45, 49, 99, 125, 126), although Espinosa-Martos et al. noted no effect of pasteurization on these cytokines (65). Similarly, a reduction post-HoP of interferon (IFN)-*γ* was reported (49, 125) but no effect was noted elsewhere (45). Interestingly, an increase in the concentration of IL-7, IL-8, and monocyte chemotactic protein (MCP)-1 following pasteurization has been recorded (45, 49, 65, 99). However, no increase in IL-8 and MCP-1 was reported (65, 125), although high levels of IL-8 retention occurred (89%) (125).

**Table 1:** Influence of Holder Pasteurization on the cytokine content of donor human milk.

Cytokine	Effect	Volume pasteurized (mL)	Pooled (number of donors)	Reference
IL-2	–	119	Yes (4)	(49)
	–	<10	No	(65)
	**↓** (47%)	<50	No	(125)
IL-4	–	119	Yes (4)	(49)
	–	<10	No	(65)
	**↓** (>70%)	<50	No	(125)
IL-5	–	119	Yes (4)	(49)
	–	<10	No	(65)
	**↓** (>45%)	<50	No	(125)
IL-12	–	20	Yes (6)	(45)
	–	119	Yes (4)	(49)
	–	<10	No	(65)
	**↓** (42%)	<50	No	(125)
IL-13	–	119	Yes (4)	(49)
	–	<10	No	(65)
	**↓** (>90%)	Not specified	No	(99)
IL-17	–	20	Yes (6)	(45)
	–	<10	No	(65)
MIP-1β	**↓**	<10	No	(65)
EPO	**↓**	Not specified	No	(126)
IL-1β	**↓**	119	Yes (4)	(49)
	**↓** (>70%)	<50	No	(125)
	–	<10	No	(65)
IL-6	**↓** (75%)	20	Yes (6)	(45)
	**↓**	Not specified	No	(99)
	**↓** (>50%)	<50	No	(125)
	–	<10	No	(65)
IL-10	**↓** (24%)	20	Yes (6)	(45)
	**↓**	119	Yes (4)	(49)
	**↓** (>75%)	<50	No	(125)
	**↓**	Not specified	No	(126)
	–	<10	No	(65)
	–	Not specified	No	(99)
TNF-α	**↓** (95%)	20	Yes (6)	(45)
	**↓**	119	Yes (4)	(49)
	**↓**	Not specified	No	(99)
	**↓** (>80%)	<50	No	(125)
	–	<10	No	(65)
IFN- γ	**↓**	119	Yes (4)	(49)
	**↓** (>60%)	<50	No	(125)
	–	20	Yes (6)	(45)
IL-7	**↑**	<10	No	(65)
IL-8	**↑** (41%)	20	Yes (6)	(45)
	**↑**	119	Yes (4)	(49)
	**↑**	Not specified	No	(99)
	–	<10	No	(65)
	–	<50	No	(125)
MCP-1	**↑** (62%)	20	Yes (6)	(45)
	–	<10	No	(65)

(↑) A significant increase in cytokine level occurred.

(↓) A significant reduction in cytokine level occurred.

(–) No significant effect on cytokine concentration was observed.

(%) The percentage reduction or increase is noted in brackets.

Similarly, HoP has variable effects on milk GFs (see Table 2). No effect of HoP on the following GFs has been reported, Transforming GF (TGF)-β1, TGF-β2, and epidermal GF (EGF) (65, 99, 126, 127). In contrast, reductions in hepatocyte GF (HGF), insulin-like GF (IGF)-1, IGF-2, IGFBP-2, and IGFBP-3 were recorded (49, 127), while increased levels of granulocyte–macrophage colony-stimulating factor (GM-CSF) following pasteurization were reported (65). It appears as though the effect of HoP on both cytokines and GFs is molecule-dependent. The mechanism by which cytokines and GFs are impacted by HoP is unknown but may be dependent on their protein structure. Espinosa-Martos et al. (65) attributed changes in concentration to their release from cellular or fat compartments into the aqueous fraction following heat exposure.

**Table 2:** Influence of Holder pasteurization on the growth factor composition of human milk.

Growth factor	Effect	Reference
TGF- β1	–	(99, 126)
TGF- β2	–	(65, 99)
EGF	–	(99, 126, 127)
HGF	**↓**	(49)
IGF-1	**↓** (39%)	(127)
IGF-2	**↓** (10%)	(127)
IGFBP-2	**↓** (19%)	(127)
IGFBP-3	**↓** (7%)	(127)
GM-CSF	**↑**	(65)

(↓) A significant reduction in growth factor level occurred.

(–) No significant effect on growth factor concentration was observed.(↑) A significant increase in growth factor occurred.

Overall, findings indicate a vulnerability of some cell signaling molecules to thermal pasteurization conditions; however, due to the limited and contrasting data a total effect cannot be determined. Due to the essential role of these molecules in the inflammatory response, minor alterations may influence the immunoprotective value of DHM. Further evaluation of changes in the balance of pro- and anti-inflammatory cells following HoP is necessary, as a shift could significantly alter the clinical value of DHM.

#### 2.3.6 MicroRNA

Human milk is a rich source of microRNAs (miRNAs), defined as short (19–24 nucleotides), non-coding segments of RNA, that act as post-transcriptional regulators of gene expression (128). miRNAs are located within extracellular vesicles called milk exosomes. Milk exosomes encapsulate miRNAs, protecting them from degradation by RNases, digestion, or low pH, facilitating their transport to target cells and tissues via the bloodstream (115, 129). Although the extent of miRNA activity is still under investigation, evidence suggests a role in cell functionality and modulation of genes involved in physiological processes, such as metabolism, neurocognitive development, and immune function (90, 130).

There have been relatively few studies assessing the impact of HoP on miRNAs. A recent study noted that miRNA, within whole milk material and milk exosomes, undergoes significant degradation as a result of HoP (131). Smyczynska et al. demonstrated an 82-fold decrease in whole material miRNA reads and a 302-fold decrease in miRNA exosome reads following HoP. This significant reduction prevented the group from further analyzing and characterizing the effects of HoP on the miRNA composition and function in DHM. However, in a subsequent study, Lamberti et al. observed a significant modification in the diversity of miRNA content of HoP-treated milk exosomes, noting 33 differential miRNAs (132). The differential miRNAs were implicated in five key pathways associated with immune and cellular function, highlighting the potential for alterations to the immunomodulatory activity of DHM as a result of processing. As it stands, the impact of HoP on the miRNA composition of DHM remains relatively unexplored. Further investigations are required to establish the full effect of HoP, however, based on the findings to date a shift in the abundance and diversity of miRNA as a result of HoP has been observed.

#### 2.3.7 Milk hormones

Milk hormones are non-nutritive bioactive compounds (69). More recently, links between hormones and infant health and development, particularly in metabolic health, have been reported (133, 134).

A small number of studies have assessed the response of milk hormones to HoP treatment. Marousez et al. demonstrated a significant effect of HoP on a variety of metabolic hormones. They reported a decrease of 63, 41, 11, 100, 41, and 83% in insulin, nesfatin-1, cortisol, leptin, apelin, and GLP-1, respectively (69), whereas adiponectin levels remained unchanged following treatment. Ley et al. (51) also noted a reduction in insulin of 46% and, unlike the above study, a decline in adiponectin of 33%. Furthermore, melatonin, a key hormone in the regulation of the sleep/wake cycle, was unaffected by HoP (135). The potential for alterations in the levels of milk hormones as a result of processing raises concern due to their roles in energy regulation and the metabolic development of infants. The potential for long-term effects on the development and physiological function should be assessed.

Although HoP-treated milk retains a significant portion of the nutritive value of DHM, HoP also depletes a range of biologically active milk components. The loss of irreplaceable bioactive agents, including Igs, lactoferrin, and BSSL is a substantial limitation to the use of HoP. The affected components have known functional and beneficial properties for the newborn. It is apparent the thermal conditions of HoP negatively disrupt the structure of bioactive milk proteins. This shortcoming calls for the development and implementation of alternative techniques with less degradative outcomes.

### 2.4 Holder pasteurization and microbial inactivation

Although bacterial levels in milk vary, starting bacterial counts of 2.7–4.1 log_10_ CFU/mL and 2.6–5.2 log_10_ CFU/mL in DHM have been reported (65, 66). Furthermore, the predominant bacteria were of the *Staphylococcus*, *Streptococcus*, *Lactobacillus,* and *Bacillus* genera. The primary objective of HoP is to remove any pathogenic microbes that could potentially infect an infant upon ingestion. A number of studies have demonstrated the efficacy of HoP at eliminating potentially harmful bacteria in DHM, both naturally occurring and through artificial inoculation.

The efficacy of HoP against naturally occurring microbes in DHM has been determined (65, 136). Lima et al. (104) noted no aerobic or coliform growth post-HoP. Additionally, in a study by Landers et al. (137), pre-HoP culturing demonstrated that of 303 milk pools, 87% were colonized with coagulase-negative *Staphylococcus*, 16% with *Enterococcus*, 8% with *α*-*Streptococcus*, 4% with *S. aureus,* and 61% of samples were colonized with at least one Gram-negative rod. Following HoP, 93% of the samples showed no bacterial growth. The persistent bacterial growth was mainly *Bacillus* growth (5%) and also coagulase-negative *Staphylococcus* (1%). This study emphasizes the need for mandatory pasteurization due to the prevalence and variability of bacterial growth in DHM. Moreover, the results to date indicate the efficacy of HoP in eliminating the non-spore-forming bacterial community of DHM. Indeed, Czank et al. assessed the capacity of HoP to deactivate inoculated bacterial strains (93). HoP was carried out on milk samples with a concentration of 1×10^5^ CFU/mL of five bacterial strains (*S. aureus*, *Enterobacter cloacae*, *Bacillus cereus*, *Staphylococcus epidermis,* and *Escherichia coli*). The tested concentration of bacteria is the maximum advisable level of growth in DHM, according to NICE guidelines (24). The study demonstrated that HoP reduced the number of bacterial species by at least 99.9%. Furthermore, the group determined the most to least heat-resistant strains were as follows: *S. aureus*, *B. cereus*, *E. cloacae*, *E. coli*, and *S. epidermis*. Similarly, findings by Raptopoulou-Gigi et al. (138) noted the ability of HoP to eliminate bacterial growth in samples inoculated with 2×10^7^ of *S. aureus* and *E. coli*.

The ability of HoP to eliminate a high concentration of non-spore-forming bacteria has been established, but an ongoing concern for milk banks is the presence of bacterial spores. A number of studies demonstrated the inefficiency of HoP in eliminating bacterial spores, particularly *B. cereus* (65, 66). In a study of 21 DHM samples, 14% of samples had *B. cereus* growth post-pasteurization (66). Indeed, the enhanced growth of *Bacillus* following exposure to thermal pasteurization conditions has been observed. Landers et al. reported *B. cereus* growth in 17 of 303 pooled milk samples post-HoP (137). Of the 17 samples, 10 were positive for *B. cereus* growth pre-pasteurization; however, the remaining 7 were only positive subsequent to HoP processing. Similarly, Lima et al. (104) reported *B. cereus* growth in 3 of 12 samples post-HoP. Again, pre-HoP culturing noted only one sample positive for *B. cereus* prior to pasteurization. These findings suggest that HoP is not only inefficient at eliminating *B. cereus* growth but also poses a risk of increasing *B. cereus* levels by inducing sporulation during thermal processing. Clearly, post-processing, DHM is at increased risk of *B. cereus* growth.

The starting levels of bacterial growth in DHM and the potential for bacterial spore contamination emphasize the need for routine pre-HoP bacterial screening. However, any post-pasteurization screening must be carried out with caution to reduce the risk of further contamination (24). It is evident that although HoP is effective in eliminating the majority of bacterial growth, this does not extend to robust organisms such as spores, in this case, *B. cereus*. This is a key challenge facing the milk banking sector, which is largely reliant on strict post-processing handling and storage protocols to minimize the risk of bacterial sporulation. The inactivation of microbial spores and other thermo-tolerant organisms should be considered when future studies evaluate the efficacy of HoP or undertake the development of alternative processing techniques.

## 3 Alternative thermal techniques

As a result of the degradative effects of HoP on bioactive agents of milk and the inability to fully eradicate bacterial spores, a variety of other thermal processing techniques are under investigation. These methods alter the processing conditions applied (temperature and duration of exposure) or the mode of thermal application.

### 3.1 High-temperature short-time pasteurization

High-temperature short-time (HTST) pasteurization is an established, effective, and efficient method of pasteurization in the commercial dairy industry (139, 140). This method requires the rapid heating of milk to 72–75°C for a short period of time (5–15 s), usually using continuous-flow plate heat exchangers. In recent years, HTST has emerged as an alternative to HoP, due to its ability to eradicate the microbes of DHM. However, it remains to be established if a reduction in the length of thermal exposure better preserves the nutritional and bioactive factors of DHM.

#### 3.1.1 HTST and the nutritional composition of DHM

HTST conditions (72.5°C/15 s) applied to DHM, with either a laboratory-scale continuous-flow device or water bath immersion, did not significantly affect the macronutrient content of the DHM (39, 122). In addition, no significant changes in the fatty acid profile or levels of malondialdehyde, an indicator of lipid oxidation, were observed (122). Further studies noted the retention of the total protein content of DHM (74, 75, 102) and a better-retained protein profile than HoP-treated milk following HTST (118). Moreover, Silvestre et al. reported a superior retention of the mean lysine concentration following HTST than HoP, 85.11 and 70.69%, respectively (75), suggesting improved protein quality with HTST-treated DHM. Again, these findings highlight the possibility that milk processing techniques may modify the nutritional quality of DHM to varying degrees despite retaining the overall nutrient levels.

Few studies have examined the effect of HTST on the vitamin micronutrient composition of DHM. The retention of folic acid and some vitamins, including vitamins B1, B2, B6, B12, and C, was reported following HTST (72°C /5–15 s) (74, 141). In contrast, Martysiak-Żurowska et al. (122) noted a reduction in vitamin C of 50.2%. However, despite the observed reduction in vitamin C, the total antioxidant capacity (TAC) of milk was maintained (122, 142).

In conclusion, HTST processing maintains the macronutrient composition of DHM. Moreover, findings suggest that HTST may be superior at retaining the protein profile and quality of DHM. The full effect of HTST on the micronutrients in DHM is yet to be established due to the limited number of studies. However, findings propose some B vitamins and the TAC are retained with HTST treatment. Further studies examining a wider range of vitamins and indeed minerals should be carried out to allow for comparison with HoP-treated DHM.

#### 3.1.2 HTST and the bioactive factors in DHM

Reports on the Ig content of DHM following HTST are limited, with the majority of data only reporting on the IgA and sIgA content of DHM. A number of studies noted high levels of retention. Indeed, following HTST (72°C/5–15 s) retention of IgA of 74–84% and sIgA of 79–100% was reported (74, 102, 141, 143), although a depletion in IgA of 36–57% was also documented (97, 144). In addition, a reduction in IgG of 42% and IgM of 100% was noted following HTST at 72°C for 15 s (97). The evidence suggests that the Ig content of DHM may incur losses following HTST; however, the extent of IgA reduction when compared with HoP suggests that HTST is superior for IgA retention. Clearly, further investigation on the IgG and IgM content following HTST is warranted.

Contrary to the findings of Goldblum et al. (141), a considerable decrease in lactoferrin levels, of 42–73%, following HTST has been widely reported (102, 122, 143). The observed reductions in lactoferrin are likely influenced by the form of lactoferrin and therefore the degree of iron saturation (122, 145). Indeed, the iron-depleted (apo) form of lactoferrin denatures at temperatures higher than 70°C at a pH of ∼7.0 (145). Taken together, the evidence suggests lactoferrin is not retained following HTST.

Several studies demonstrate the maintenance of lysozyme concentration and activity following HTST (102, 122, 146), and two studies reported increased lysozyme activity (141, 144). However, a reduction in lysozyme levels of 80% was reported elsewhere (74). The contradictory findings may be caused by different methods of biochemical analysis. For instance, studies noting lysozyme retention used turbidimetric *M. lysodeikticus* assays (122, 141, 144) or liquid chromatography–mass spectrometry (LC/MS) (102), whereas Hamprecht et al. employed radial immunodiffusion to evaluate lysozyme levels (74). Overall, the evidence supports the stability of lysozyme during HTST. As discussed previously, lysozyme is heat labile at the pH of milk (113), suggesting the shorter exposure time of HTST processing prevents the destruction of lysozyme.

Similar to HoP, a significant depletion in human milk lipase, particularly BSSL, occurs following HTST (74, 141, 146). Kontopodi et al. noted low retention of BSSL levels and activity of 9 and 19%, respectively (102). Nonetheless, the observed reductions in HTST-DHM are significantly less than those of HoP-treated DHM (102, 146). In addition, the digestive enzyme *α*-amylase underwent a reduction in activity of 7% following HTST (122). The complete destruction of ALP following HTST has been repeatedly demonstrated, confirming the efficacy of HTST pasteurization conditions (74, 102, 143).

Studies on the influence of HTST on cell signaling molecules are sparse. However, a reduction in IGFBP-2 and the retention of IGF-1, IGF-2, IGFBP-3, and EGF have been demonstrated (127). These findings are in contrast to HoP, where in the same study the authors noted a significant reduction following HoP in all of the GFs analyzed, except EGF (127).

The contents of lactoferrin, lysozyme, and Ig of DHM are likely contributors to the intrinsic bacteriostatic ability of milk. Indeed, a negative correlation between a reduction in these three proteins and the growth rate of bacteria was identified (102). Thus far, findings are in agreement that HTST impacts the bacteriostatic capacity of milk, with increased levels in the growth rate of *E. coli* in HTST-treated DHM in comparison with raw milk (102, 147), although no differences in the growth of *S. aureus* were observed between raw and HTST-DHM (102). It remains unclear, whether HTST or HoP treatment has a more degradative influence, due to contrasting findings. However, the effect of HTST on the bacteriostatic capacity of milk against *E. coli* is apparent. Further studies are needed to determine which processing technique best preserves the bacteriostatic properties of milk. A depletion in the natural bactericidal capacity of DHM following processing is concerning, as those in receipt of DHM are at increased risk of infection and disease. Minimizing the negative effect of thermal processing on the natural bacteriostatic capacity of raw milk should be considered when selecting an ideal technology for milk processing.

Findings suggest that HTST is superior or at the least equivalent to HoP at retaining some of the bioactive agents of milk. The observed variability in results may be due to the holding time and temperature applied, the chosen method of biochemical analysis, or the apparatus used for HTST processing. For instance, a wide assortment of methodologies has been used to perform HTST including plate heat exchange (141), water bath immersion (122, 144), and various laboratory-scale continuous-flow devices (74, 143, 146). Indeed, the apparatus used to implement HTST may have differing effects on the temperature and cooling profiles of DHM. In particular, temperature distribution may vary with non-continuous-flow instruments, resulting in portions of the sample being exposed to higher thermal conditions for increased periods. Evidently, precise temperature regulation, calibration of instruments, and a uniform approach are critical to gather comparable findings.

#### 3.1.3 Microbial inactivation with HTST

The ability of any milk processing technology to effectively eliminate bacterial growth is critical in order to be considered for a milk bank setting. HTST successfully inhibits the naturally occurring microbial community in DHM (123, 141, 146). The microbial deactivation of DHM following HTST has been further demonstrated with inoculation studies. Bacterial spiking studies demonstrated the efficacy of HTST against *E. coli* (10^8^ CFU/mL), *S. aureus* (10^7^–10^8^ CFU/mL), *Streptococcus agalactiae* (10^6^ CFU/mL), *Listeria monocytogenes* (10^9^ CFU/mL), and *Cronobacter sakazakii* (10^9^ CFU/mL) (146, 148). However, a critical finding is that HTST was not effective against naturally occurring *B. cereus* (123). In this study, HTST conditions of 70–75°C for 5–25 s were tested and none of the temperature–time combinations applied were capable of *B. cereus* eradication. For HTST to be a favorable alternative to HoP, inactivation of *Bacillus* is advisable. Further studies should assess the potential resistance of *B. cereus* to HTST conditions.

Overall, HTST appears equally effective to HoP at eliminating the microbial community of DHM; however, both procedures fail to address the ongoing challenge of *B. cereus* contamination. Furthermore, although widely adopted by the dairy industry, the logistics of implementing HTST in a milk bank setting may be challenging. Aside from high costs, HTST equipment requires cleaning between each pasteurization cycle, making it labor-intensive. Additionally, in comparison with the dairy sector, much smaller volumes are processed by milk banks. Although some of these issues may be overcome, Giribaldi et al. (146) designed a small-scale continuous-flow HTST instrument with the ability to process 0.1–10 L of milk. Such a device would need to be readily available commercially and undergo further testing. Importantly, the need for cleaning between cycles is still required. Evidently, aside from the need for further studies, the practicality and feasibility of downscaling HTST for clinical use warrants further consideration.

### 3.2 Retort processing

Retort processing is a commercial food sterilization technique that applies heat (115–121°C) and pressure (15–20 psi) to a product for a set holding time before subsequent cooling (149). Recently, retort processing has been utilized for the preservation of DHM, producing a shelf-stable product that is currently available to neonatal intensive care units in the USA (104).

In all studies to date, retort processing of DHM was carried out at 121°C, at 20 psi, for 5 min using water immersion (40, 104, 150, 151). Retort processing preserved the gross macronutrient composition of DHM (40). However, a reduction in lysine and thiamine of 20 and 80%, respectively, was observed. As noted previously, a reduction in lysine could indicate a modification in the protein quality, despite no observed change in total protein levels. A significant reduction in bioactive proteins of milk following retort processing occurs in lactoferrin, IgA, IgG, and IgM of 84% (150, 151), 88–100% (104, 150, 151), 77–100% (150, 151), and 100% (150), respectively. In addition, lysozyme was reduced by 54–89% (104, 151), although the retention of lysozyme was reported elsewhere (150). Furthermore, retort processing results in almost the complete degradation of vascular endothelial GF (VEGF) and TGF-β2, whereas TGF-β1 is maintained (150). A clear advantage of retort processing is the complete microbial inactivation of DHM, including *B. cereus* (104).

However, it is apparent that the efficiency of microbial inactivation is at a significant nutritional cost. The bioactive properties of DHM are at risk of almost complete destruction. A further advantage associated with retort processing is the ability to be packaged and subsequently stored at room temperature where cold storage facilities are lacking. Despite these advantages, the nutritional and therapeutic value of shelf-stable DHM is significantly diminished and may require fortification post-processing, suggesting it is an inferior alternative to HoP-treated DHM.

### 3.3 Microwave heating

Despite the small number of studies, microwave heating has emerged as a promising novel pasteurization method. Microwave heating relies on the generation of microwaves, which penetrate the food product, where they are rapidly absorbed and dissipate energy in the form of heat (152).

Leite et al. assessed the influence of microwave heating at 60°C, 65°C, and 70°C for 30 s, 15 s, and 10 s, respectively, on certain bioactive factors in DHM (153). The group noted that at all temperature and time combinations, microwave heating did not alter the concentration of oligosaccharides or fatty acids in DHM. Furthermore, microwave heating at 60°C for 30 s maintained 82, 88, 95, and 87% of IgA, IgG, IgM, and lactoferrin, respectively. Similarly, no significant reduction in the Ig or lactoferrin content was observed following processing at 65°C for 15 s. However, microwave heating at 70°C for 5 s did cause significant reductions in IgG, IgM, and lactoferrin levels of 37–76%. Comparable results were reported following microwave heating at 62.5°C for 5 min (154). High levels of retention were observed for lactoferrin, sIgA, and vitamin C of approximately 60-92%. Furthermore, the retention of TGF-β2 was documented. However, the study noted a significant depletion of approximately 91% in basal lipase activity following microwave heating. In addition, a reduction in lysozyme concentration and activity of approximately 56 and 30%, respectively, occurred.

These findings demonstrate the potential of microwave heating in milk banks with high levels of bioactive factors retained. However, the choice of temperature and length of treatment is critical. Indeed, when Martysiak-Żurowska et al. increased the temperature of microwave heating to 66°C for 3 min, a significant loss in all of the bioactive components was observed (154). These findings indicate that microwave heating at 60–65°C allows for optimal retention of bioactive factors of DHM when the treatment duration is short (15–30 s), whereas an increased treatment time of 5 min may deplete levels of lysozyme and lipase.

More recently, when microwave heating with HTST conditions of 72.5°C for 15 s was applied the macronutrient content and fatty acid profile of DHM were maintained (122). Furthermore, the levels of lactoferrin and lysozyme were unchanged post-processing. However, a reduction in vitamin C and *α*-amylase of 42.6 and 6%, respectively, was observed. The retention of lactoferrin and lysozyme is in contrast to previous findings at 66°C for 3 min and 70°C for 10 s (153, 154). The contrast in findings calls for further assessment of microwave heating at increased temperatures (>66°C) on DHM to allow for an optimal time–temperature combination to be determined.

Critically, the ability of microwave heating at 60°C for 30 s to eradicate the natural microbiota of DHM and inactivate ALP has been demonstrated (124). Moreover, microwave heating achieved a complete reduction of *Salmonella typhimurium* and *S. aureus* inoculated at concentrations of 10^6^ CFU/mL (124). Despite the encouraging findings, the data on the capacity of microwave heating for microbial inactivation are lacking. Further assessment of the inhibition of thermo-resistant microbes and the pasteurization conditions applied is needed. Once the optimal treatment parameters for microbial inactivation have been established, subsequent analysis on the macro- and micronutrients of DHM should be performed.

Overall, findings to date on microwave heating are encouraging but preliminary. A key advantage of microwave heating is its ease of operation and accessibility within a milk bank setting. Furthermore, microwave heating requires little space and is time-saving, cost-effective, and low energy. However, the non-uniform transfer of heat during microwave heating may pose a challenge, resulting in the creation of cold spots in the milk (152). Further evaluation of the dielectric properties of human milk and its subsequent interaction with microwave heating is required to ensure consistent results. Indeed, a stirring mechanism or the equivalent should be implemented to prevent this from occurring. In addition, strict monitoring of time and temperature is essential.

### 3.4 Thermo-ultrasonication

The combination of ultrasonication (20–100 kHz) and moderate heat (>50°C) to induce bacterial inactivation in foodstuffs is known as thermo-ultrasonication (TUS). Ultrasonication occurs following the formation and cavitation of microscopic bubbles. Bubbles are produced as a result of pressure shifts, following ultrasound wave propagation during processing. These bubbles collapse rapidly and with force, producing heat and shockwaves. The consequence of this is cellular membrane disruption and subsequent cell death (155).

Thus far, the limited data indicate that the bioactive properties of DHM undergo moderate reduction following TUS, the extent of which is mainly determined by ultrasound power (W) and also exposure time. For instance, the treatment of DHM at 40 W for 9 min maintained the IgA, lactoferrin, and lysozyme content, although a reduction in BSSL was observed (101). In comparison, when the power was increased to 60 W for a reduced period of 6 min, TUS retained IgA and lactoferrin but reduced lysozyme and BSSL by more than 40 and 70%, respectively (101). Moreover, following TUS at 60 W for 9 min, a significant reduction in all the examined bioactive proteins occurred and levels of retention were comparable to those observed post-HoP (101). As indicated by the authors, the degree of bioactive protein retention decreased as the power of ultrasound increased. In a separate study, high levels of insulin retention (97%) have been reported, with a lower retention rate of 67% following HoP (156). It appears that TUS has the potential to better preserve some bioactive properties of DHM; however, this is likely dependent on the choice of power, time, and temperature applied.

The ability of TUS to cause microbial deactivation of inoculated strains of *S. epidermis*, *E. coli,* and *S. aureus* has been previously observed (157, 158). Indeed, Kontopodi et al. demonstrated the ability of TUS (40–60 W, 6–9 min) to cause a > 6-log CFU/mL reduction of inoculated *E. coli, S. epidermis, and E. cloacae* in DHM (101). These findings were further supported by Mank et al. (156) who recorded a 5-log reduction in the same bacterial strains following TUS at 60 W for 6 min. Overall, TUS appears an effective technique for the microbial inactivation of DHM, with significant reductions of inoculated pathogenic strains reported to date. However, the efficacy of TUS against bacterial spores is yet to be determined. In particular, further evaluation of TUS at 40 W is advisable, due to the optimal retention of bioactive proteins observed at this pasteurization condition.

Uniquely, TUS presents the opportunity to reduce thermal exposure of DHM and consequently may produce superior DHM. Evidently, although the findings to date are encouraging, additional studies are required to establish the efficiency of TUS and identify the optimal treatment parameters for DHM pasteurization. In particular, the macro- and micronutrient composition of DHM following TUS remains unknown.

### 3.5 Experimental temperature and time durations

A number of studies have carried out pasteurization at a range of temperatures and holding times to assess the effects on the nutritional and microbial components of human milk. Due to the experimental processing conditions chosen, these experiments do not fit within the parameters of previously discussed thermal techniques and are presented below, categorized according to thermal exposure. The results from these studies may provide greater insight into the optimal conditions for thermal processing of DHM.

#### 3.5.1 Use of temperatures <62.5°C

Various studies reduced the pasteurization temperature or holding time below that of HoP (<62.5°C, <30 min). Czank et al. reported a 90% retention of sIgA, lactoferrin, and lysozyme following processing at 57°C for 30 min with an experimental pasteurizer (93). Similarly, pasteurization at 40–55°C for 30 min retained >60, >80, and > 80% of IgA, lactoferrin, and lysozyme, respectively (159). The observed difference in protein retention, despite the lower thermal conditions of the latter study, may be due to the pasteurizer design. The experimental pasteurizer used by Czank et al. was capable of precise temperature and time regulation.

In contrast, Zhang et al. used a bottle immersion technique. Although this method more closely reflects the technique applied in milk banks, it does not have the same capacity for temperature monitoring. Both groups concluded that the temperature applied during processing, not the length of exposure, was the critical factor for protein retention. Indeed, a 1°C increase in temperature caused a reduction in the retention of all three proteins; however, a proportionate reduction in exposure time had no effect (93). Moreover, processing at 62.5°C for 5 s resulted in a much lower retention of lactoferrin (32%) and lysozyme (72%) but a similar retention of IgA (83.2–95%) (143, 160). These findings further suggest that the temperature applied during pasteurization is the key factor influencing bioactive protein retention.

Importantly, exposure to temperatures >50°C reduced the endogenous bacteria in DHM (3.15–4.78 log_10_ CFU/mL) to undetectable levels (159). Indeed, pasteurization at 57°C for 30 min was sufficient to eradicate inoculated bacteria at a concentration of 1 × 10^5^ CFU/mL, including *B. cereus* (93). In contrast, bacterial growth persisted following processing at 62.5°C for 5 s (143, 160). Additionally, ALP retention of 6–27.3% suggests that 5 s of treatment is inadequate for DHM pasteurization. Collectively, the findings demonstrate the inactivation of bacterial contamination and the improved retention of bioactive properties of DHM when processed at 55–57°C for 30 min. Further examination of microbial inactivation at these processing conditions is merited. In particular, the inclusion of heat-resistant bacteria is necessary, as studies of treatment at higher temperatures for longer periods have noted the persistence of *Bacillus* spores (66, 104, 123). The clinical potential of the improved retention of bioactive factors needs to be balanced with microbial safety.

#### 3.5.2 Treatment at 80–100°C

Multiple experiments with high-intensity treatment conditions of 80–100*°C* have been performed. As a whole, findings demonstrate the poor preservation of bioactive factors at this increased temperature range. Low levels of retention of 0–22% in IgA, IgG, and IgM were observed following treatment at 80–100°C (97, 143, 144). Similarly, a reduction of 78–85% in lactoferrin occurred when processed at 81–88°C for 5 s and levels were undetectable following 100°C for 5 min (97, 143). Lysozyme demonstrated the greatest resistance to thermal exposure, with no significant reduction following processing at 80°C for 15 s (144). However, when the length of treatment increased to 10 min, a ∼ 25% reduction was observed, which further increased following processing at 90°C (144).

Not surprisingly, the microbial inactivation of DHM occurs following pasteurization at high temperatures. A > 5-log reduction in *C. sakazakii* and *L. monocytogenes* and a > 3-log reduction in *Enterococcus faecalis* were achieved with pasteurization at 81.5°C for 5 s (143).

Evidently, DHM processing at increased holding temperatures results in a significant decrease in the key bioactive components of DHM. Moreover, at higher temperatures, the length of exposure can further exacerbate the observed degradation. This is evident with the level of lysozyme retention decreasing with an extended processing time, despite the same temperature being applied (144). Overall, there is no demonstrated benefit to the treatment of DHM at these high-temperature conditions. A similar microbial inactivation has been achieved with lower temperatures, allowing for greater retention of bioactive components of milk.

#### 3.5.3 Treatment >100°C

Heating temperatures of >100°C are commonly applied in the dairy industry, e.g., in cases of sterilization (∼121°C) or ultra-high temperature (UHT) (135–150°C) (161, 162). The extreme temperatures used by the dairy industry, when applied to DHM, have an expected negative impact on bioactive properties of milk. Indeed, less than 20% of IgA and lactoferrin were retained following pasteurization at 127°C for 15 s (159). Similarly, following UHT, the Ig content of DHM was undetectable and an 84% reduction in lactoferrin occurred (150). Again, lysozyme was the most resistant to high-temperature processing, with approximately <40% retention following 127°C for 15 s (159) and no observed reduction following UHT (142°C, 6 s) (150).

Furthermore, an effect that has not been seen at lower processing temperatures is the impact of sterilization conditions on the lipid fraction of DHM. Processing at 120°C for 30 min decreased the available lipid content by ∼10% due to the formation of surface skin and deposit on the inside of the sterilization container (36). Additionally, sterilization reduced the fatty acid content of DHM, specifically linolenic and arachidonic acid (36). Whether these effects occur at sterilization and UHT temperatures, with a reduced processing time, is not known. The degradation of bioactive properties of DHM and its potential influence on the lipid fraction emphasize that the current techniques used by the dairy industry are not suitable for adoption by milk banks.

## 4 Non-thermal processing techniques

Accumulating evidence illustrates the degradation of various DHM components with prolonged thermal exposure. The destruction of the heat-labile components produces DHM with a reduced therapeutic value. As a result, various non-thermal strategies are now being assessed for DHM pasteurization.

### 4.1 High-pressure processing

In recent years, high-pressure processing (HPP) has been shown as a safe, effective, and alternative method of preservation in the food industry. HPP is widely used in a number of foodstuffs, including vegetable, meat, and dairy products, due to its ability to reduce microbial load while retaining high nutritional value and protecting the organoleptic characteristics of foods (163, 164). Furthermore, HPP-treated food is viewed as minimally processed, lending to a high level of consumer acceptability (165). During HPP, food is vacuum-packed and placed into a pressure vessel containing a pressure-transmitting fluid. It is then subjected to isostatic pressure, typically 400–600 MPa at 4–45°C (164). Pressure is delivered instantaneously and distributed evenly throughout the product. The primary adjustable conditions that influence the efficacy of HPP are the pressure selected (MPa), the holding time, and the temperature (166). As pressurization does not disrupt covalent bonds, the loss of nutrients and bioactive compounds is minimal. Instead, pressure impacts weaker bonds (Van der Waals forces, electrostatic interactions, and hydrogen bridges) resulting in a loss of cellular function, membrane disruption, and subsequent microbial inactivation (165).

#### 4.1.1 HPP and nutritional composition

Findings to date indicate that HPP at 300–600 MPa for 5–10 min does not cause any change in the macronutrient content of DHM (41, 42, 167). Despite this, a 2.9% reduction in total carbohydrate levels following HPP at 500 MPa for 8 min was reported (41). However, further supporting the stability of the carbohydrate portion of DHM, Marousex et al. (80) noted the retention of HMOs following HPP at 350 MPa. Similarly, a reduction was reported in some n-3 series PUFAs following HPP for 6 min at 600 MPa (45). However, others note the retention of the lipid profile of DHM following HPP (300–600 MPa), with no reported changes to the fatty acid composition or the triacylglycerol profile (43, 44, 47, 167). Furthermore, following HPP, the total protein profile of DHM was unchanged (76) and more closely resembled that of raw milk than HoP-treated milk (78). Overall, these findings indicate the retention of the macronutrient composition of milk following HPP.

The influence of HPP on the micronutrient content of DHM has been limited to vitamin composition, with varying findings. Tocopherols underwent significant reductions following HPP at 600 MPa of 21–27%, 44–47%, and 25–33%, in *α*-, *δ*-, and *γ*-tocopherol, respectively (45), whereas pressurization at 400 MPa retained α- and δ-tocopherol but reduced γ-tocopherol by 26–29% (45). In contrast, a separate study reported the retention of tocopherol levels following HPP at 400–600 MPa for 5 min (44). Similarly, contradictory reports of vitamin C stability have been recorded. Despite a reported reduction in vitamin C content of 75% following HPP at 500 MPa for 8 min (41), the retention of vitamin C following processing at 400–600 MPa for 5 min has also been demonstrated (44). Folate was unchanged by HPP at 500 MPa for 8 min (41). In addition, one study has reported on the carotenoid content of DHM; both *β*-carotene and lycopene, important anti-oxidants, are preserved following HPP at 450–600 MPa for 10–15 min (47). However, lutein and zeaxanthin underwent reductions of ∼60%.

Collectively, the findings suggest that the effect of pressurization on the vitamin composition of DHM is distinct for each vitamin. Furthermore, the extent of vitamin reduction seemingly increases with an increase in pressure. The impact of holding time remains unclear but may account for the observed discrepancy in vitamin C retention. It is evident that an investigation of a wider variety of vitamins and indeed minerals is required to fully ascertain the influence of HPP on the micronutrient composition of DHM and any subsequent clinical significance.

#### 4.1.2 HPP and bioactives

To date, there is promising evidence of Ig retention following HPP. Indeed, a number of studies noted the retention of IgG, IgM, and IgA following HPP at 200–450 MPa for 2.5–30 min (94, 96, 98, 101, 168). However, a significant depletion in Ig is observed with an increased pressure of 600 MPa (76, 94, 96, 101, 144, 169). For instance, a reduction in IgA, IgM, and IgG of 20–26%, 59–60%, and 35–40%, respectively, occurred subsequent to HPP at 600 MPa for 15–30 min (98). Although a reduced holding time of 2.5 min maintained IgA levels, losses of 21% in IgM and IgG still occurred. Clearly, the choice of pressure and exposure time are influential factors.

This was further illustrated by Permanyer et al., who noted that, as pressure increased, an additional reduction in IgA occurred; 100% retention at 400 MPa, 87.9% at 500 MPa, and 69.3% at 600 MPa (94). Similarly, Kontopodi et al. (101) observed a greater reduction in IgA levels with increased treatment time during HPP at 600 MPa, with 55% retention after 3 min and 47% after 5 min. On the whole, these findings suggest that HPP can retain the Ig profile of DHM but the selection of processing parameters is a critical determinant. In particular, as the pressure or time exposure increases, so too does the risk of Ig loss. Further studies should ascertain the optimum holding time for HPP treatment, with findings to date suggesting that HPP at <500 MPa is suitable for Ig retention.

The retention of lactoferrin following HPP at 350–600 MPa with assorted holding times has been demonstrated (76, 101, 170, 171). Conversely, a reduction of 25% following HPP at 500 MPa for 8 min was reported (41). Similar reductions of 35–44% were observed following HPP depending on the combination of pressure, holding, and resting times applied (169). Importantly, the optimal variant group tested, HPP at 200 MPa for 10 min followed by a 10 min resting period and 400 MPa for 10 min (200 + 400 MPa), did not cause a significant reduction in lactoferrin. Evidently, the HPP specifications applied will influence the degree of lactoferrin denaturation. For example, following 15 min of HPP at 300, 400, 500, or 600 MPa, a reduction in lactoferrin of 9, 23, 34, and 48%, respectively, occurred (110). Furthermore, the majority of reduction in lactoferrin occurred within the first 5 min of treatment, with only a further 8% loss incurred between 7 and 30 min of treatment (110). Despite the varying outcomes, the evidence suggests the superior retention of lactoferrin following HPP as opposed to HoP treatment (41, 169, 171). Unlike HoP, which results in the aggregation of disulfide bonds and denaturation of lactoferrin, HPP does not impact covalent bonds (110, 171, 172), thus better preserving the lactoferrin content of DHM. Overall, HPP can maintain lactoferrin, but this is dependent on the pressure applied and to a lesser degree, the holding time.

Although lysozyme is more robust to thermal processing than other bioactive proteins, a degradative effect of heat treatment has been observed (98, 168). In contrast, HPP at 200–600 MPa at various holding times of 2.5–30 min retains the lysozyme levels of DHM (41, 98, 101, 168, 170, 171). Collectively, the available data substantiate the stability of lysozyme during the HPP of DHM.

The influence of HPP on cell signaling molecules is still in its infancy with a small number of studies performed and contrasting data gathered. The content of cytokines and growth factors in DHM following HPP is presented in Table 3. In brief, there is consensus on the stability of IL-6, IL-8, IL-12, and TNF-α following HPP at 400 MPa (45, 99), despite a reported reduction in IL-10 of 31–42% following HPP at 400–600 MPa (45). Franch et al. only noted a reduction in IL-10 when a pressure of 600 MPa was applied (99). Similar disparate findings on TNF-*α* retention were reported following HPP at 600 MPa (45, 99). A reduction of 22–33% in monocyte chemotactic and activating factor occurred following HPP at 400–600 MPa (45). In addition, IFN-*γ* and IL-17 underwent varying losses, depending on the time–pressure combination applied (45).

**Table 3:** Influence of high-pressure processing on the levels of cell signaling molecules in human milk.

	Effect	Processing conditions	Reference
**Cytokine**			
IL-6	–	400–600 MPa, 3–6 min	(45, 99)
IL-8	–	400–600 MPa, 3–6 min	(45, 99)
	**↑**	600 MPa, 5 min	(99)
TNF-α	–	400–600 MPa, 3–6 min	(45, 99)
	**↓**	500–600 MPa, 5 min	(99)
IL-10	**-**	400–600 MPa, 3–6 min	(45, 99)
	↓	400-500 MPa, 5 min	(99)
MCAF	**↓** (22–33%)	400–600 MPa, 3–6 min	(45)
IFN-γ	**↓** (90–95%)	400–600 MPa, 3 min	(45)
	–	400–600 MPa, 6 min	(45)
IL-17	–	400 MPa, 3 min	(45)
		600 MPa, 6 min	
	**↓** (84–100%)	400 MPa, 6 min	(45)
		600 MPa, 3 min	
**Growth Factor**			
HGF	**↓** (64%)	600 MPa, 10 min	(167)
	**↓** (61%)	100 MPa + 600 MPa	(167)
	**↓** (57%)	200 + 600 MPa	(167)
	–	200 + 400 MPa	(167)
	**↓** (21%)	450 MPa, 15 min	(171)
EGF	–	400–600 MPa, 5 min	(97)
TGF-β1	–	400–600 MPa, 5 min	(97)
TGF-β2	–	400–600 MPa, 5 min	(97)

(↑) A significant increase in level of cytokine/growth factor occurred.

(↓) A significant reduction in level of cytokine/growth factor occurred.

(–) No significant effect on cytokine/growth factor concentration was observed.

(%) The percentage reduction/increase is noted in brackets.

A similar variation in GF retention following HPP has been recorded. For instance, HGF levels were either retained or underwent a reduction of 57–64% following HPP depending on the conditions applied (169). Furthermore, a 21% reduction in HGF levels occurred following HPP at 450 MPa for 15 min (173). The retention of EGF, TGF-β1, and TGF-β2 following HPP has been recorded (99). Evidently, HPP has variable effects on the cytokine and GF levels of DHM, depending on the HPP conditions applied and the cell signaling molecule in question. It is apparent from the conflicting data that further research is needed to determine the appropriate conditions for cytokine and GF retention. However, the highest retention levels across the widest range of molecules appear to be HPP at 400 MPa for <5 min. It is unlikely that a complete lack of effect on cell signaling molecules is achievable. However, HPP with optimal pasteurization parameters may retain key molecules involved in the inflammatory process and ensure a preferable balance of pro- and anti- inflammatory molecules.

Only one study to date has evaluated the miRNA portion of DHM following HPP treatment. Smyczynska et al. noted that, unlike HoP, HPP did not decrease the miRNA reads of DHM (131). Nevertheless, HPP did result in changes to the composition of the miRNA fraction. In particular, reduced levels of miRNA-29 and miRNA-148a-3p were observed. On the other hand, miRNA-30d-5p appeared highly resistant to pressurized conditions. The authors suggest a protective effect of milk exosomes during pressurization. The consequences of compositional changes on miRNA functionality and subsequent immune responses are not known. The limited available data suggest that HPP may be less detrimental to the miRNA content of human milk than HoP. However, the modification of DHM miRNA following milk processing and the potential implications this may have for the recipient requires further study.

The degradative effect of thermal processing on human milk lipases has been widely reported. Therefore, the demonstrated potential of HPP to retain high levels of milk lipases is an encouraging prospect. Wesolowska et al. reported a 79–87% retention of milk lipase activity following HPP at 450 MPa and 200 + 400 MPa (47, 173), whereas HPP at 600 MPa resulted in <17% retention in lipase activity (47). However, further supporting the maintenance of milk lipase following HPP, LPL was preserved following HPP at 400 MPa for 5 min (171). Furthermore, BSSL is retained following exposure to pressures of 400–600 MPa for 5 min (171), 8 min (41), and 1.5–30 min (101).

The influence of HPP on the hormonal constituents of DHM indicates the retention of some key metabolic hormones. Indeed, both leptin and insulin were retained when processed at 350 MPa (69), 450 MPa (173), and 200 + 400 MPa (169). Similarly, nesfatin-1, cortisol, and GLP-1 were stable after processing at 350 MPa (69). In contrast, HPP resulted in a depletion of adiponectin and apelin of 50–85 and 20%, respectively (69, 169, 173). It remains unclear why adiponectin and apelin appear more vulnerable to pressurization. However, as hypothesized by Marousez et al., it may be a result of pressure-induced modifications to both the physiochemical properties of milk and the structural composition of apelin and adiponectin (69). Despite these observed reductions, the overall hormonal composition of DHM appears well preserved following HPP, in particular, the higher levels of leptin and insulin when compared with HoP-treated DHM.

Despite the modification in the levels of some bioactive factors in DHM, the bacteriostatic ability of milk is preserved following HPP (101, 170, 174). Indeed, DHM processed at 350 MPa retained its antimicrobial activity when tested against *E. coli* and *S. agalactiae* (170). Similar findings were observed following HPP at 400–500 MPa and HPP at 200 + 400 MPa against *S. aureus* and *E. coli* (101, 174). In contrast, a higher pressurization of 600 MPa for 3–5 min did reduce the inhibition rate of DHM against *S. aureus* (101). These findings suggest that DHM processed at <500 MPa preserves the bacteriostatic capacity of DHM.

#### 4.1.3 HPP and microbial inactivation

Multiple studies have demonstrated the ability of HPP at 400–600 MPa to reduce the endogenous bacteria of DHM to undetectable levels (42, 94, 168, 171, 175). Importantly, HPP at 300 MPa for up to 15 min did not eliminate the native bacterial population of DHM, deeming it unsuitable for application within the milk bank setting (171, 175). Further studies have assessed the bacterial inactivation of inoculated pathogens following HPP. The first inoculation study was performed by Viazis et al., who reported an 8-log reduction in *L. monocytogenes* and *S. agalactiae*, after HPP at 450 MPa for 2 and 4 min, respectively (95). The group also demonstrated a 6–8 log reduction in *E. coli* and *S. aureus*; however, 30 min of treatment was required. These findings indicate the potential for pressure-resistant microorganisms.

However, subsequent studies have reported the inactivation of *S. aureus* and *E. coli*, following HPP. For instance, 15 min of HPP at 500 MPa was sufficient to reduce *S. aureus* by 5-log CFU/mL (175). In addition, a complete reduction in *S. aureus*, *E. coli*, *L. monocytogenes*, *C. sakazakii*, and *B. cereus* was recorded after HPP at 450 MPa for 15 min, with a starting concentration of 10^5–7^ CFU/mL (173). Furthermore, a > 7.8-log reduction in *S. epidermis* and *E. cloacae* was reported following HPP at 400–600 MPa for 5 min (101). Clearly, despite the pressure-resistant capacity of some microbes, when optimal HPP parameters are applied, microbial deactivation is achieved. Moreover, a 5-log reduction is the maximum necessary reduction if the acceptable growth limits according to milk banking guidelines are followed (24). However, the available data on the microbial safety of DHM following HPP remain limited in comparison with the extensive studies carried out on HoP-treated DHM. Accordingly, future studies should further endeavor to confirm the capacity of HPP at 400–600 MPa to eradicate microbial growth and determine the minimum treatment time required particularly focusing on those strains with demonstrated pressure resistance and bacterial spores, mainly *B. cereus*.

#### 4.1.4 Novel HPP conditions

Alternative approaches to HPP of DHM have been tested through an optimized HPP system (176) or by combining pressure treatment with extreme temperatures (177, 178). Martysiak-Żurowska et al. combined HPP at 193 MPa with a sub-zero temperature of −20°C (177). The group found that HPP at −20°C did not alter the FA composition or the concentration of secondary lipid oxidation products. Furthermore, the total vitamin C and TAC levels of DHM were preserved, although a reduction in ascorbic acid of 11% was observed. The authors hypothesize that extreme temperatures can intensify the effect of pressurization, allowing a lower pressure to be applied. This study presents the potential for the application of lower pressures, enabling greater preservation of bioactive components of milk. However, the data are preliminary and must be further substantiated—in particular, the capacity of lower pressure at sub-zero temperatures to not only retain the nutritional and bioactive content of milk but also ensure microbial inactivation. In contrast, a study that combined pressure with high temperatures (178) found that treatment at 65°C and 80°C depleted the *α*-tocopherol, fatty acid composition, and some cytokines, regardless of the pressure applied. Furthermore, only a pressure of 300 MPa at 50°C resulted in moderate Ig retention of 48–100%. Evidently, thermal HPP results in disruption to the nutritional and bioactive components of DHM.

Separately, an optimized, novel HPP system was applied to DHM (176). The group designed a HPP system in which the parameters for pressure delivery are accounted for, including compression rate, decompression rate, and application mode. The process parameters were as follows: pressure (350 MPa), temperature (38°C), application rate (1 MPa/s), and application mode (four cycles of 4 min duration with 5 min latency time between each cycle). Following processing, high levels of sIgA, lactoferrin, and lipase were retained at 63–64%, 93–97, and 80%, respectively. Critically, the group demonstrated a 6-log CFU/mL reduction of inoculated *S. aureus* and *B. cereus*. The potential of this optimized HPP system to eradicate bacterial spores emphasizes its potential for milk pasteurization. Indeed, 10% of donated milk samples in this study were contaminated with *B. cereus* (176). This approach would produce milk high in nutritional value with improved microbial safety. Implementation of this HPP system should be performed in future studies with further assessment of microbial inactivation and an evaluation of its influence on the nutritional composition of milk.

### 4.2 UV-C irradiation

Ultraviolet (UV)-C irradiation is an emerging innovative alternative technique for milk pasteurization. UV-C (200–280 nm) exposure disrupts the cell’s genetic material, interfering with the cell’s ability for DNA transcription and replication, resulting in cell death (101). The penetrative ability of UV-C during milk processing faces two challenges. First, the high absorption coefficient of milk due to its opaque appearance (300 cm^−1^ at 254 nm). In comparison, translucent solutions such as water have a much lower absorption coefficient (0.1 cm^−1^) (179, 180). Second, the total solid concentration of milk, which varies, can further increase the absorption coefficient (181, 182). Despite these challenges, the ability of UV-C to penetrate bovine milk has been achieved by creating a thin film turbulent flow-through of milk, ensuring exposure of the whole sample to the UV source (180, 182, 183). A similar approach has been adopted by studies assessing UV-C processing of DHM.

Only a small number of studies have reported on the macronutrient content of DHM following UV-C. Pitino et al. reported no significant changes in the concentration of carbohydrates, lipid, and protein content of DHM following UV-C treatment (41). Furthermore, UV-C at 4863 J/L caused no significant changes to the protein concentration and fatty acid profile of DHM, except for an increase in the level of fatty acid C8:0 (101, 179). However, increased levels of lipid oxidation end products following UV-C at a low dosage of 172.9–740 J/L have been reported (184). Similarly, there has been minimal reporting on the micronutrient composition of DHM following UV-C irradiation. Two studies have recorded reductions in vitamin C and folate of 35–72 and 25%, respectively (41, 184). Despite this reduction, the TAC of DHM was preserved (184). Overall, the macronutrient composition of UV-C treated DHM appears stable post-processing. However, the observed increase in lipid oxidation end products calls for further evaluation of the lipid fraction post-processing. Additionally, any potential loss or alteration in the concentration or composition of micronutrients remains relatively unknown.

As this process is non-thermal, heat-induced denaturation of bioactive compounds does not occur. Studies suggest that UV-C preserves key immune factors including lactoferrin, lysozyme, and IgA. Lactoferrin retention of 90–95% and 80–87% following UV-C at 2084–3645 J/L and 4,683–4,863 J/L, respectively, has been reported (101, 103, 181). Similarly, high levels of lysozyme preservation were observed at 84–96% and 75–80%, respectively (101, 103, 181), although lower levels of lysozyme retention (∼60%) were reported elsewhere (184). Studies of the Ig profile following UV-C are limited and have focused on the IgA content. IgA levels were preserved at >80% following UV-C at 2430–4863 J/L (101). These findings were further supported by sIgA retention of 89–95% following UV-C at 2084–4683 J/L (103, 181). Clearly, findings suggest that UV-C may be a suitable non-thermal alternative to HoP that will better preserve key DHM components. Indeed, comparative studies noted much lower levels of retention of lactoferrin, lysozyme, and IgA following HoP of 9–32%, 41–78, and 40%, respectively (101, 103, 181). In addition, a number of studies have reported the retention of BSSL following UV-C at 1,100–5,500 J/L (101, 119, 179). Indeed, a reduction in BSSL of 64.7% was only observed when a high UV-C dosage of 16,500 J/L was applied, which further increased to 79.8% at a dosage of 33,000 J/L (119). These findings demonstrate that BSSL is stable when exposed to moderate UV-C exposure.

The retention of immune proteins and antioxidant factors is a likely contributor to the preserved bacteriostatic activity of UV-C-treated DHM. Barbarska et al. noted the preserved bactericidal capacity of DHM following UV-C at 6720 J/L. Indeed, untreated DHM caused a 46.6% reduction in *E. coli* growth, whereas, following UV-C irradiation, *E. coli* growth was reduced by 57.6–62.6%. In comparison, HoP-treated DHM only reduced growth by 12% (174). Retention of the antimicrobial activity of DHM following UV-C at 2084–4863 J/L against *S. aureus* and *E. coli* was also demonstrated elsewhere (101, 103). These findings suggest that UV-C retains not only the bioactive properties but also the subsequent antimicrobial activity of milk.

A number of studies have demonstrated the efficacy of UV-C in ensuring sufficient microbial inactivation of DHM. Christen et al. were the first to note that UV-C at a dosage of 4,836 J/L results in a 5-log CFU/mL reduction of *S. epidermis*, *S. aureus*, *E. cloacae,* and *B. cereus* (179). Similarly, at the same dosage, a reduction of 5.78–6.95 log CFU/mL was achieved in DHM inoculated with a concentration of 10^8^ CFU/mL *S.* e*pidermis*, *E. cloacae,* and *E. coli* (101). In the same study, a reduced UV-C dosage of 3,645 J/L and 2,430 J/L resulted in log reductions of 4.6–5.9 and 4.3–5, respectively (101). These findings were further supported by Almutawif et al. (181) who observed a > 5-log reduction in *S. aureus* when UV-C was applied to DHM at 2259 J/L. In fact, no growth persisted following treatment for 14 days at 4°C and 18 h at 37°C.

However, a separate study reported that a higher dosage of 2,750 J/L resulted in a less than 5-log reduction in *C. sakazakii*, *E. faecium*, *L. monocytogenes,* and *S. aureus* (119). Furthermore, this dosage only resulted in a 2.75 log reduction in bacterial spores of the *Bacillus* and *Paenibacillus* species. The study noted that a high UV-C dosage (8,250 J/L) was required for a > 5-log reduction across all strains tested, including spores. Finally, UV-C treatment at 85–740 J/L is inadequate for microbial inactivation of DHM, with reductions in *E. faecium* of <1-log following 550 J/L and < 3-log following 740 J/L (119, 184). Evidently, UV-C processing of DHM can produce microbiologically safe milk when the correct parameters are applied. Findings thus far indicate that a UV-C dosage of 4,863 J/L and 8,250 J/L can inactivate the microbial components of DHM, including bacterial spores.

Although there have been positive indications for UV-C treatment at lower doses (2259–3,645 J/L), further studies are necessary due to the contradictory results reported. In particular, the potential survival of bacterial spores requires further examination. Furthermore, prior to processing the total solid concentration of the milk samples should be quantified. As observed by Christen et al., the required UV-C dosage applied to milk increases linearly with the total solid concentration of milk (179). This may account for the discrepancies noted in findings to date, in addition to the applied turbulent flow rate and pasteurizer design during UV-C processing.

As a whole, the research suggests that UV-C at >4,863 J/L results in a > 5-log reduction in microbial growth, the maximum allowed growth in milk banks (24). Importantly, significant retention of the bioactive properties of milk is also achieved with these treatment parameters, as summarized in Table 4. The observed variance in bacterial reduction at lower dosages suggests further study is required to ascertain the minimal parameters for UV-C treatment of milk. All studies discussed applied a turbulent flow to milk while undergoing UV-C to expose DHM evenly and overcome the high absorption coefficient of milk. To optimize treatment parameters, future studies should assess the total solid content of milk prior to processing. In addition, further evaluation of the influence of UV-C on other key factors with biologically active roles, including IgG, IgM, cytokines, and hormones, is warranted.

**Table 4:** Percentage retention of bioactive proteins following UV-C processing of milk.

Bioactive compound	UV-C (J/L)	Retention (%)	Reference
Lactoferrin	2,084	95	(103)
	2,259	92	(181)
	2,430	>90	(101)
	3,474	93	(103)
	3,645	>85	(101)
	4,683	87	(103)
	4,863	>80	(101)
Lysozyme	85	100	(184)
	740	∼60	(184)
	2084	91	(103)
	2,259	96	(181)
	2,430	>85	(101)
	3,474	84	(103)
	3,645	>80	(101)
	4,683	75	(103)
	4,863	>80	(101)
IgA^*^	2084	95	(103)
	2,259	92	(181)
	2,430	>90	(101)
	3,474	94	(103)
	3,645	>90	(101)
	4,683	89	(103)
	4,863	>80	(101)

* Includes IgA and sIgA.

### 4.3 Gamma irradiation

Gamma irradiation is a non-thermal technology that can be used to pasteurize foodstuffs. During processing, the product undergoes controlled exposure to gamma rays from a radioactive source, for example, cobalt-60 or cesium-137. The gamma rays are absorbed by the food product, damaging the genetic material of microbial cells, inducing cell death, and consequently pathogen reduction (185–187). Despite the approval of gamma irradiation use in certain food groups by multiple agencies, including the WHO, the European Food Safety Authority, and the US Food and Drug Administration (188–190), consumer hesitancy persists (191). Consequently, studies of this technology for DHM pasteurization are minimal and its applicability to milk banks may be viewed unfavorably. Gamma irradiation exposure of up to 10 kGy is sufficient to ensure microbial safety, retain the nutritional components of foodstuff, and limit radiolysis (185, 187). Consequently, 10 kGy has mostly been applied as the maximum dose in DHM studies to date and the results are presented herein.

Gamma irradiation of DHM has mainly been applied as part of a hybrid approach with either freeze-drying or the addition of an antimicrobial mixture. For instance, Blackshaw et al. studied the effects of gamma irradiation on freeze-dried DHM. The group suggest that 2–5 kGy is the optimal exposure range for the retention of milk’s nutritional properties (192). Indeed, irradiation at 2 kGy retained the protein profile of DHM and did not increase the levels of lipid oxidation end products. In a separate study, the same group demonstrated the capacity of irradiation at 5 kGy to completely eliminate strains of *S. aureus* and *S. typhimurium* inoculated at a concentration of 10^6^ CFU/mL (193). Furthermore, the bacteriostatic properties of DHM were retained following irradiation (193). Similarly, Balaji et al. (194) used an alternative hybrid approach on DHM of gamma irradiation at 5 kGy combined with antimicrobial formulations. The hybrid treatment resulted in a 6-log reduction in bacteria, including *B. cereus*. Furthermore, the combined treatment maintained the Ig and lactose levels in DHM. However, protein hydrolysis and a subsequent increase in peptide concentration occurred; while this did not impact the *in vitro* digestibility of protein.

Few studies have assessed the potential of gamma irradiation as a standalone treatment. That being said, reported reductions of 70.8, 18, and 28% in BSSL, IgA, and lactoferrin, respectively, occurred following gamma irradiation of DHM (119, 138). Importantly, both studies applied a high dosage of 25 kGy during processing. The increased dosage may explain the depletion in IgA, as opposed to its retention following hybrid IR processing. However, the levels of BSSL and lactoferrin have not been assessed elsewhere, and therefore, the influence of a reduced irradiation dosage remains unknown.

Clearly, there is not enough data to determine the efficacy of irradiation for DHM processing. Further evaluation of the influence of gamma irradiation on the nutritional, bioactive, and microbial composition of DHM is required. Future studies should be carried out at a dosage of approximately 5 kGy to ensure optimal retention and allow for comparison with more recent findings. Overall, based on findings to date and potential consumer perspective issues, gamma irradiation is an unlikely candidate for milk bank implementation. However, the initial results highlight the value of combined hybrid treatments. Indeed, the combination of gamma irradiation and freeze-drying results in effective microbial inactivation and retention of key antimicrobial and nutritional properties of DHM. Advantageously, this hybrid approach would allow for the pasteurization of pre-packaged powdered DHM with an extended shelf life. Additionally, the use of antimicrobial formulations may contribute to increased shelf stability of DHM. The applicability of freeze-drying or antimicrobials in combination with alternative processing technologies should be further explored.

### 4.4 Pulsed electric field treatment

Pulsed electric field (PEF) treatment is a non-thermal novel processing technique that results in bacterial inactivation whilst preserving the nutritional and sensory characteristics of food (195). During PEF, the food is placed between two electrodes, and short high-voltage (approximately 20–80 kV/cm) pulses are applied (195). Consequently, pore formation occurs in the microbial cell membrane, this is known as electroporation (196). The structural changes to the cell membrane as a result of electroporation cause an imbalance in cell homeostasis and induce cell death (197). To date, only two studies have explored PEF processing of DHM. Sanchaya et al. (198) demonstrated the retention of the physiochemical properties and the lipid content of DHM following PEF. In addition, PEF at 35 kV for 3,000 μs was sufficient to eliminate the naturally occurring microbes of DHM. In contrast, bacterial growth persisted following processing at 25–30 kV for 3,000–4,500 μs.

Separately, Zhang et al. performed nanosecond (ns)-PEF on DHM (199). The group used response surface methodology, a mathematical modeling system, to determine two optimum processing conditions for DHM. These were 15 kV voltage with 6,000 pulses at a frequency of 20 Hz or 50 Hz. Indeed, following nsPEF at 20 Hz, retention of lysozyme, sIgA, and lactoferrin of 76, 108, and 74%, respectively, was observed. Moreover, nsPEF increased the levels of xanthine oxidase and a greater than 60% retention of the enzyme lactoperoxidase was observed.

Of the sparse data available, the processing of DHM with PEF has encouraging results. However, it is evident that substantial research is still required to fully establish its effect prior to its application in a milk bank setting. Nonetheless, the accomplished studies point to the potential processing parameters for future investigations.

## 5 Discussion

Human milk processing is an area of research that has undergone much investigation and progress in recent years. In particular, alternative pasteurization technologies have emerged with the potential for implementation by the milk bank sector. The emerging technologies can be categorized into two groups: thermal and non-thermal processing techniques.

Heat treatment of food is a long-established method for food preservation. Indeed, the concept of heating milk before infant feeding was first suggested in 1824, and by 1889, heat-treated milk dispensaries were conceived (200). Currently, HoP is the widely accepted milk processing technique established within milk banks. However, in recent years, the negative influence of HoP on valuable bioactive components of DHM has been substantiated. Consequently, the appraisal of substitute thermal processing techniques has been explored. Of these, HTST has been the most extensively studied. Despite the potential superior retention of some DHM components, the effect on milk lipases and lactoferrin is degradative. Other components, such as cell signaling molecules and the bacteriostatic capacity of milk, remain unclear. A potential drawback of HTST is its logistical applicability to a milk bank setting.

In contrast, thermal processing by microwave heating has greater accessibility and is less labor-intensive. However, data on this experimental technique remains scarce and an overall impact on DHM components cannot yet be determined. Similarly, although findings on TUS processing are positive, the research is still in its infancy and further studies are necessary to substantiate the results to date on novel technologies and to determine the optimal treatment parameters. Conversely, retort processing and pasteurization at high temperatures (>80°C) incur significant losses in various nutritional and bioactive factors, suggesting that the required conditions are too harsh for DHM processing.

Non-thermal methods have rightly generated much interest due to the protective capacity of such techniques on the heat-labile compounds of DHM. Of the non-thermal technologies, HPP stands out as the most promising and consequently most studied. Determining the optimal HPP parameters should therefore be a priority (201). However, neglected areas of research remain, mainly, the impact on micronutrient content of DHM, in particular mineral content and bioactive constituents including miRNA and milk hormones. Furthermore, in comparison with HoP, further study of microbial deactivation with pressurization is required. Although the potential of UV-C has been demonstrated, the limited existing data impedes a determination but does encourage further evaluation of this technique. Similarly, PEF and gamma IR are novel techniques with scarce findings requiring further evaluation. The applicability of these processing techniques in terms of feasibility, implementation, and consumer acceptance should be considered. Unlike newer technologies, HoP has been extensively examined; consequently, an unevenness in the volume and range of data exists. For a direct comparative analysis to be enabled, further research must be carried out on these alternative processing technologies that remain relatively unexplored. In particular, the capacity for bacterial spore inactivation should be a primary focus, as this is a major limitation of the currently favored HoP technique.

A core issue across all studies using various modes of pasteurization is the variability in study designs. To date, studies have differed vastly in terms of pre-processing collection, handling, and storage of samples. Furthermore, the number of freeze–thaw cycles to which a sample is exposed should be clearly presented where possible. All of these factors may influence the milk constituents and their vulnerability to processing conditions. In addition, both the sample volume and number of donors per pool have differed significantly across studies. These factors can influence the total solids concentration and the required treatment time, potentially impacting both the effectiveness of the technique, the subsequent analysis and the comparability of findings. Similarly, following processing, variation in the analytical methods deployed is common. Collectively, these alternating approaches make it difficult to discern the true effect of processing when contrasting results are reported. Recently, a review by Kontopodi et al. presents the most commonly used analytical methods and suggests a template workflow for evaluating DHM processing (83). Adherence to an accepted workflow and consideration of the aforementioned factors would increase uniformity and may reduce the variability in findings. Evidently, a standardized approach when assessing milk processing techniques would facilitate future comparative analysis.

Despite the wide range of technologies examined to date, some areas remain relatively unexplored, for instance, biopreservation. The potential use of antimicrobials in food preservation is gaining momentum in dairy and other foodstuffs (202–204). Future studies should endeavor to assess their potential in DHM, in particular, as part of hybrid processing with other technologies. Indeed, hybrid processing, as demonstrated in a small number of studies, may reduce the severity of processing conditions required. In addition, the production of DHM in powdered form may provide greater transport and storage options—in addition to prolonged shelf life. These factors are particularly important in under-resourced areas, where refrigeration storage may not be possible and milk supply is low. Another technique under investigation by both the dairy and infant formula industries is the use of microfiltration as a method of milk pasteurization. This technique is yet to be studied in DHM and should be evaluated in the future. Furthermore, although beyond the scope of this review, DHM may be enhanced by fortification to replace lost nutrients—or indeed, inoculated to allow for the re-introduction of mothers’ own milk microbiota (205–210). The combination of optimal processing techniques with the potential for personalized nutrition may produce the highest value DHM.

Human milk is a highly valuable food that influences and shapes the infant’s gut, physiological development, and immune system maturation. Breast milk promotes the survival and development of the infant while ensuring tailored nutrition with an ample supply of immune proteins. The impact of alternatively processed DHM on the clinical value for the newborn recipient should be further assessed, and any processing technique under consideration should first be assessed for its efficacy in microbial inactivation. Bacterial spores present a persistent challenge that future technologies should be equipped to overcome.

In conclusion, the heterogeneity in sample history, study design, and analytical methods used have contributed to divergence in the available information for optimal processing of DHM and prevented a complete understanding of the emerging processing techniques. A uniform approach to future studies and comprehensive information regarding sample history will allow further insight and optimization of milk banking pasteurization. Most importantly, any future technology should mitigate the risk of bacterial contamination whilst promoting the optimal retention of bioactive components, ensuring DHM that confers the highest quality nutrition to the newborn recipient.
